# Immune Reconstitution Inflammatory Syndrome Presenting as Disseminated Kaposi Sarcoma

**DOI:** 10.7759/cureus.34832

**Published:** 2023-02-10

**Authors:** Gabriele Ruzgas, Shayet Hossain Eshan, Shrungavi Ramanathan, Ashwini Gotimukul, Rohan K Bodapati

**Affiliations:** 1 Family and Community Medicine, University of Illinois Chicago, Chicago, USA; 2 Internal Medicine, Ascension Saint Joseph - Chicago, Chicago, USA; 3 Internal Medicine, University of Wisconsin School of Medicine and Public Health, Madison, USA

**Keywords:** doxorubicin, antiretroviral therapies, art, iris, disseminated kaposi sarcoma, pulmonary kaposi sarcoma, kaposi sarcoma, hiv

## Abstract

We present a patient who was previously diagnosed with HIV and had multiple violaceous skin lesions at the time of his diagnosis. Following the initiation of antiretroviral therapy (ART), the number of lesions increased significantly and he developed shortness of breath, which prompted hospital admission for further workup. Biopsy of the skin lesions confirmed the diagnosis of Kaposi sarcoma (KS). Bronchoscopy with biopsy revealed KS lesions in his respiratory system. Imaging and biopsy confirmed KS invasion of lymph nodes. Due to widespread KS, he was diagnosed with immune reconstitution inflammatory syndrome (IRIS). Because of the lack of improvement on ART alone, he was started on chemotherapy, which decreased the size of existing skin lesions, stalled the development of new skin lesions, and led to symptom improvement. As a result of this case, we recommend that treatment teams have close follow-ups of patients started on ART and that they remain mindful of the possibility of IRIS. Disseminated KS may warrant a prompt response with chemotherapy to improve outcomes.

## Introduction

Kaposi sarcoma (KS) is a well-known AIDS-defining illness caused by human herpesvirus-8 (HHV-8) that has been less frequently observed since the development of antiretroviral therapy (ART) [1]. Regardless, it remains a critical malignancy to identify and treat early, given the potential for visceral involvement; this includes pulmonary KS, which has been documented to have a poor prognosis [2]. Dissemination of KS may occur in the setting of immune reconstitution inflammatory syndrome (IRIS), a condition in which there is a worsening of pre-existing conditions following initiation of ART. There are no universally accepted diagnostic criteria for the condition, therefore diagnosis typically requires a temporal relationship between ART initiation and worsening of pre-existing conditions, while still maintaining a positive response to ART. IRIS may be classified as either "unmasking," meaning an asymptomatic pre-existing condition becomes clinically evident, or "paradoxical," meaning that a symptomatic pre-existing condition clinically worsens [3]. Of note, disseminated KS in the setting or IRIS may occur regardless of the patient's CD4 count prior to initiation of ART [4]. This case will highlight the importance of screening patients for opportunistic infections prior to ART initiation, surveilling patients after ART initiation to monitor for IRIS, including pulmonary KS as a differential for respiratory symptoms in patients with HIV, and promptly considering chemotherapy for treatment of disseminated KS.

## Case presentation

Presentation

An African American non-Hispanic male in his late 20s with a past medical history of HIV, hepatitis B, and asthma was admitted for shortness of breath and diffuse skin lesions. 

Four months prior to this admission, the patient tested positive for HIV during incarceration, at which time he had already had the presence of maculopapular skin lesions, but he was not started on ART. Two months prior to this admission, he underwent repeat testing for HIV, which revealed a viral load of 115,000 copies/mL and CD4 count of 556; he was subsequently started on ART with Biktarvy (bictegravir/emtricitabine/tenofovir alafenamide). Additionally, the patient's hepatitis B viral load was 120000 IU/mL. Tests for gonorrhea and chlamydia (urine, rectal, and throat), HCV antibody, QuantiFERON-TB (QIAGEN N.V., Hilden, Germany), and cytomegalovirus (CMV) DNA polymerase chain reaction (PCR) were all negative. His most recent CD4 count one week prior to admission was 547.

**Figure 1:**
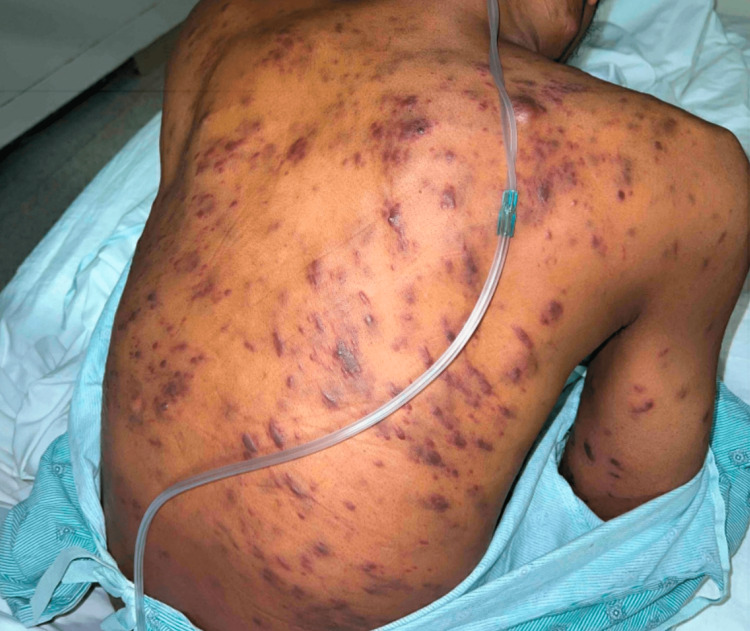
Kaposi sarcoma lesions on the patient's back.

**Figure 2:**
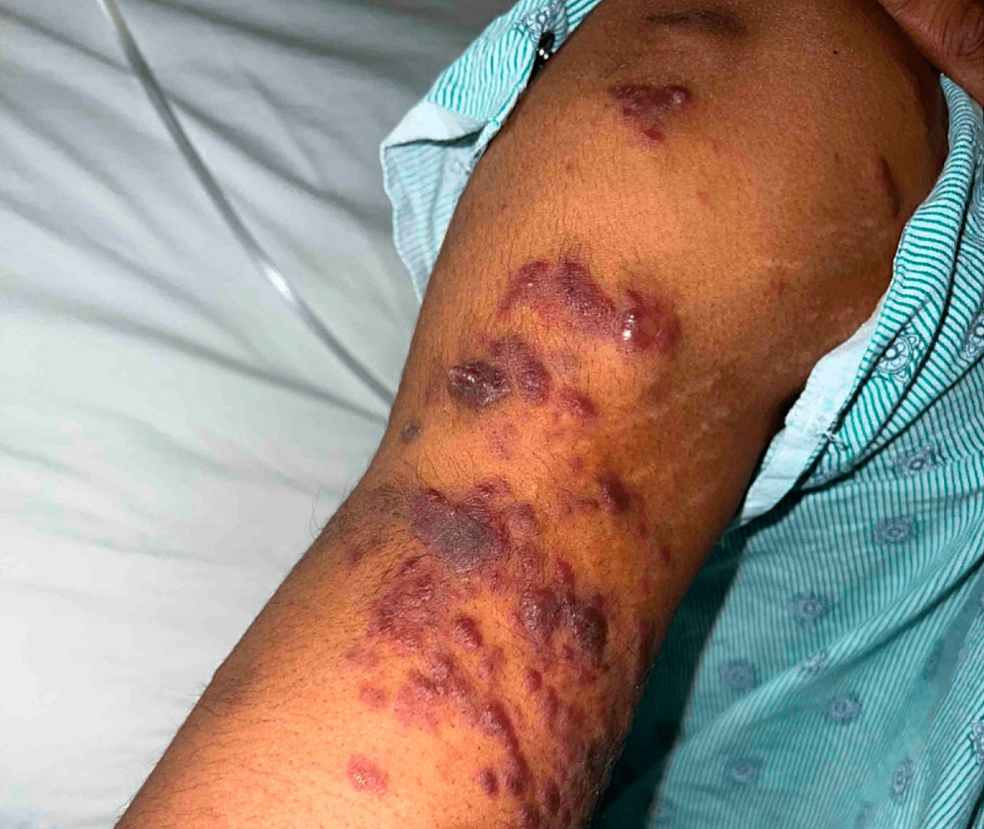
Kaposi sarcoma lesions on the patient's right upper arm.

**Figure 3:**
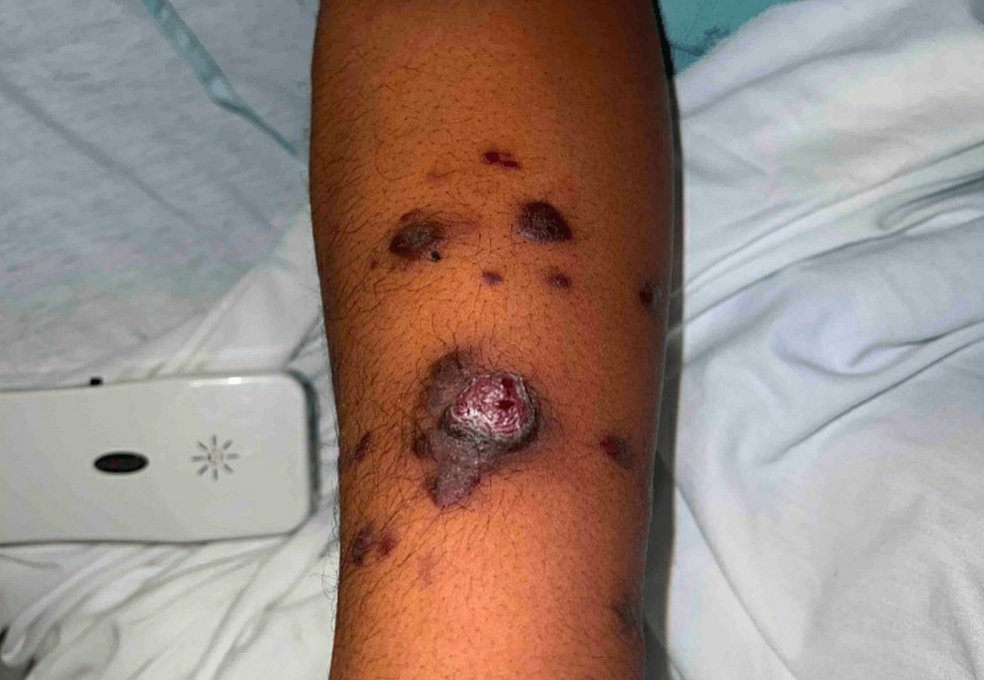
Kaposi sarcoma lesions on the patient's right lower arm.

**Figure 4:**
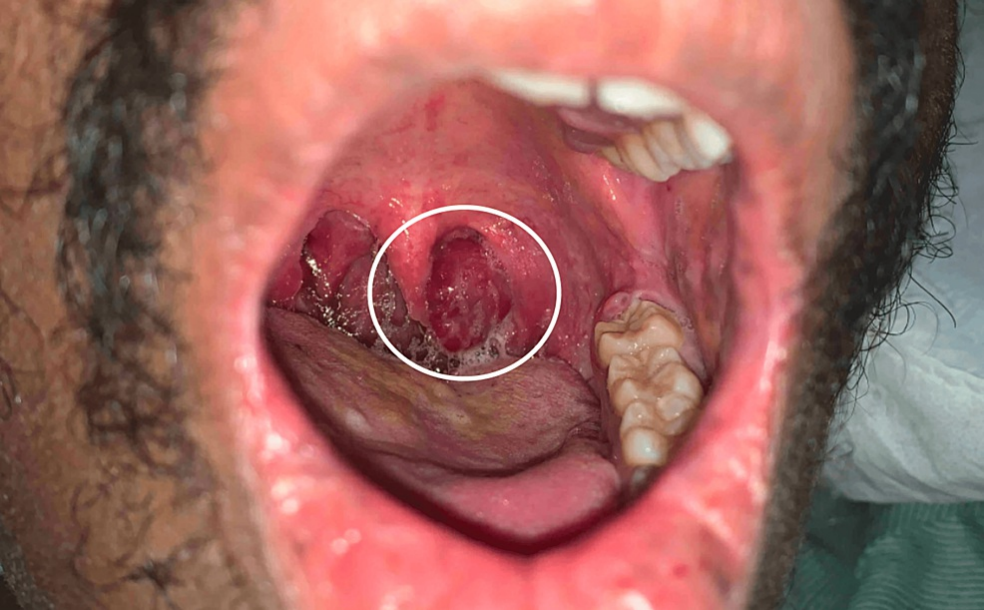
Suspected Kaposi sarcoma lesion in the left oropharynx.

Following the initiation of ART, the patient noted that his skin lesions multiplied in number. On admission, there were widespread, violaceous, painless papules, plaques, and nodules ranging in size from 2 mm up to 18 mm with some of the smaller lesions coalescing. There were also occasional vesicles overlying the papules and plaques (Figures 1-3). He also noted a prominent oropharyngeal lesion that he felt with swallowing, though it did not cause any dysphagia or odynophagia (Figure 4).

**Figure 5:**
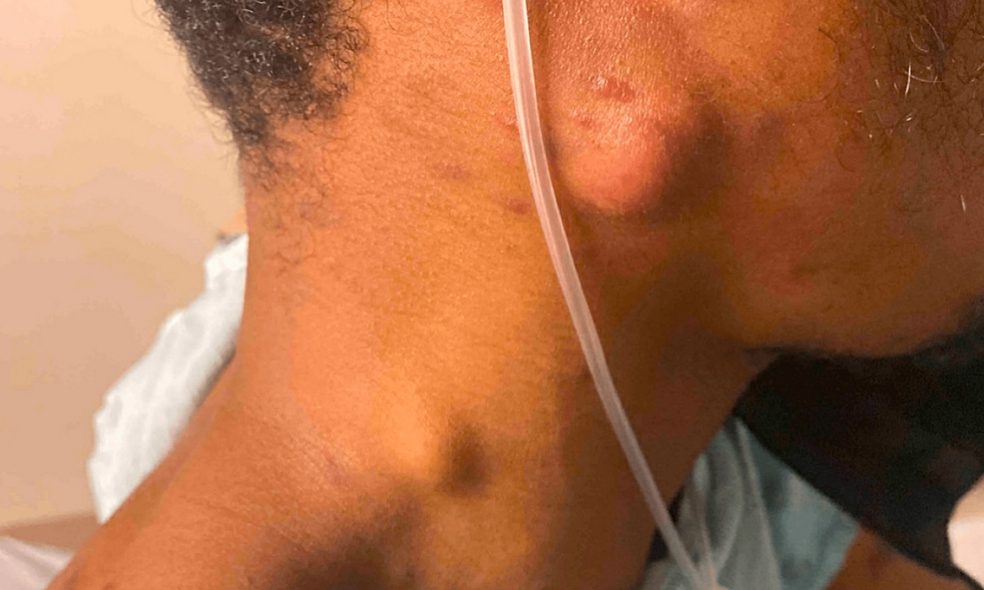
Two non-tender enlarged lymph nodes on the patient's right neck.

Additionally, he had prominent non-tender lymphadenopathy and swelling on presentation. Visual inspection revealed one firm, non-fluctuant right upper anterior cervical lymph node measuring approximately 8 mm and another firm, non-fluctuant right lower posterior cervical lymph node measuring approximately 3 mm (Figure 5). His right arm, bilateral legs, and genitalia were significantly edematous. This edema caused discomfort, although he could still ambulate and urinate appropriately.

Workup and diagnosis

**Figure 6:**
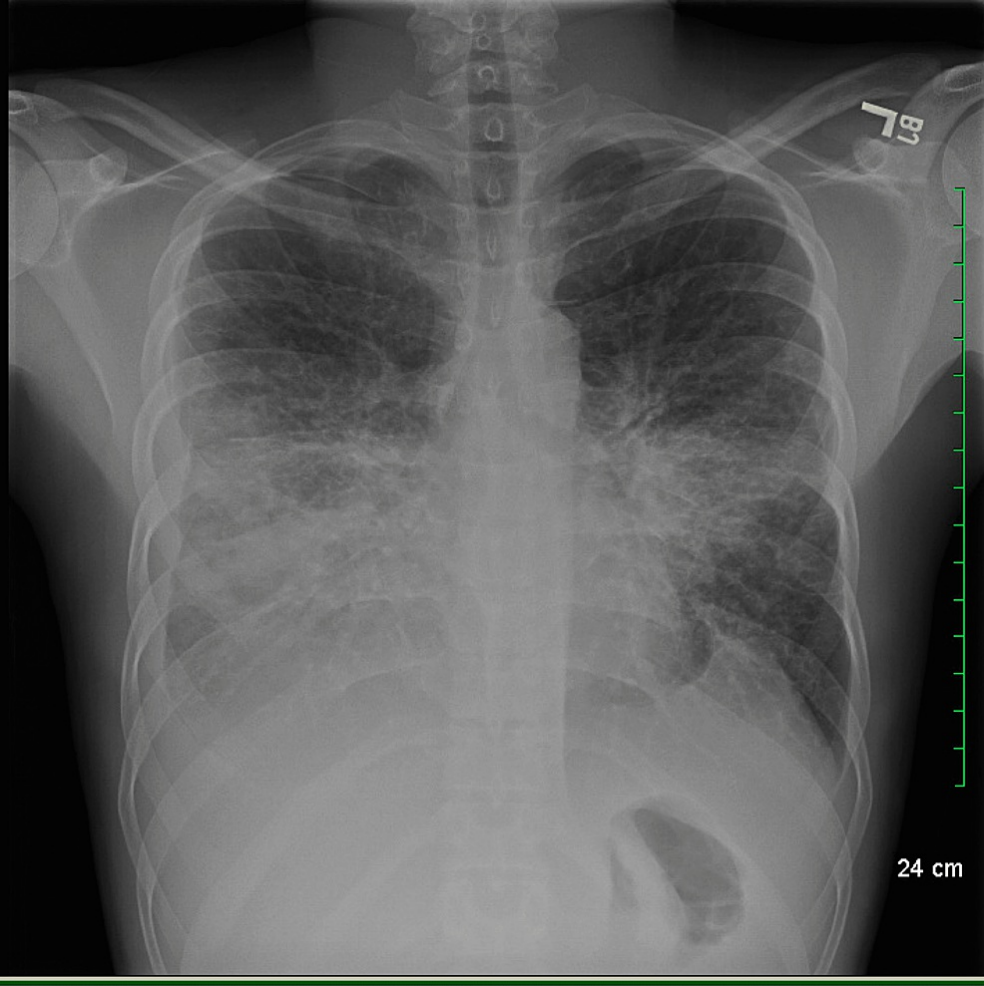
Patient's initial chest x-ray on admission.

We began prompt and concurrent workup of the patient's skin lesions, lymphadenopathy, and shortness of breath. A skin biopsy of a lesion on his left thigh was positive for HHV8, confirming the highly suspected diagnosis of KS. An initial chest x-ray revealed bilateral pneumonic infiltrates, with associated bilateral pleural effusions, worse on the right side (Figure 6). At this point, the primary team considered a number of pathogens that could cause pneumonia, including opportunistic pathogens; the differential also included concurrent pulmonary KS given the patient's widespread skin lesions and lymphoma, and Castleman disease given the patient's diffuse lymphadenopathy.

We proceeded to obtain coronavirus disease 2019 (COVID-19) PCR, respiratory virus panel, QuantiFERON-TB test, histoplasmosis antibody labs, blastomycosis antibody labs, histoplasmosis urine antigen, blastomycosis urine antigen, cryptococcal serum antigen, and bacterial and fungal blood cultures. He tested positive for coronavirus variant OC43 but was negative otherwise. The 1,3-β-D-glucan test (Fungitell®, Associates of Cape Cod, Inc., Falmouth, Massachusetts, United States) was positive, as was the repeat test several days later, despite no apparent signs of fungal infections and negative fungal workup. Therefore, we decided to defer systemic antifungals due to the possible side effects associated with this therapy. We decided to conduct thoracentesis for both diagnostic and therapeutic purposes. Pleural fluid analysis revealed negative Gram stain and culture, negative acid-fast bacteria (AFB) culture, and negative fungal culture. Cytology was negative for malignancy. However, the pleural fluid met 1/3 Light's criteria and was therefore exudative.

**Figure 7:**
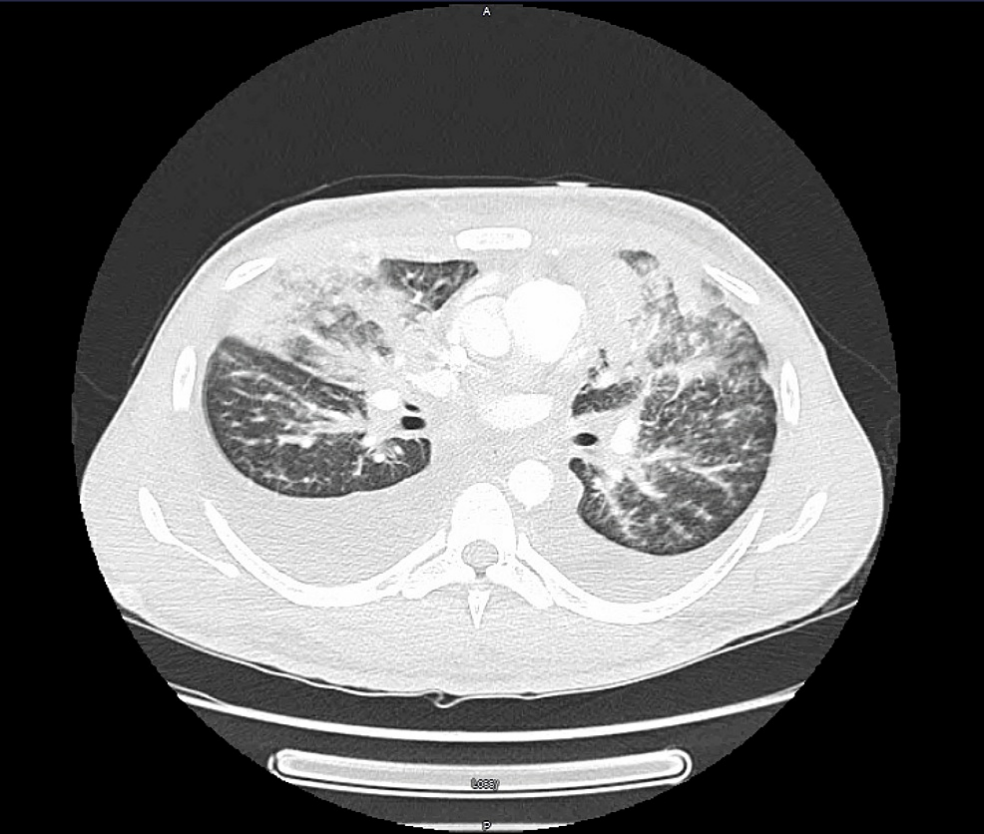
Cross-section of patient's chest CT showing ground glass opacities, air space opacities, and bilateral pleural effusions.

Following this initial workup, we planned to obtain additional imaging and perform broncho-alveolar lavage (BAL) for additional pathogen testing. Given the patient's pulmonary findings and widespread lymphadenopathy, we proceeded with CT imaging of the neck, chest, and abdomen. Chest CT showed diffuse areas of ground glass opacities throughout the lungs and bilateral pleural effusions (Figure 7). Neck and abdomen CT imaging showed enlarged and necrotic lymph nodes of the neck, a necrotic lymph node in the right axilla, mediastinal lymphadenopathy, and several enlarged inguinal lymph nodes. Imaging did not reveal any hepatic, gallbladder, pancreatic, renal, or gastrointestinal lesions. Of note, we did not obtain CT imaging of the head given the patient did not have any neurologic symptoms or neurologic deficits on exam.

Given our wide differential and CT imaging that showed significant lymphadenopathy, we decided to proceed with bronchoscopy for BAL, lymph node biopsy, and lung biopsy. BAL culture and Gram stain, AFB smear, and fungal culture were all unremarkable. The sample was also negative for legionella, aspergillus, and pneumocystis. Fine-needle aspiration via bronchoscopy of the right paratracheal lymph node revealed spindle cell proliferation and HHV8+ staining consistent with KS. Transbronchial biopsy of the right middle and upper lobes showed fibroblast-like proliferation and HHV8+ cells, consistent with KS, which confirmed a diagnosis of pulmonary KS. Of note, we did not biopsy the patient's oropharyngeal lesion per the otolaryngology team's recommendations, given the risk of the lesion being highly vascular and a biopsy possibly causing excessive bleeding or respiratory compromise. Given the patient's disseminated KS lesions, the oropharyngeal lesion remained highly suspicious of KS.

The patient's shortness of breath was most likely caused by concurrent COVID-19 infection and pulmonary KS. Given the rapid dissemination of KS lesions following the initiation of ART, while still maintaining a positive response to ART supported by the maintenance of CD4>500, the patient was diagnosed with disseminated KS secondary to paradoxical IRIS.

Treatment and outcomes

For symptomatic treatment, we administered 2-4 L supplemental oxygen via nasal cannula for shortness of breath, which achieved sufficient O_2_ saturation levels and therefore did not require escalation. We also administered loop diuretic therapy to reduce fluid volume that may contribute to lymphedema, and multimodal analgesics for pain management. He underwent thoracentesis to drain the bilateral pleural effusions. Unfortunately, this procedure was complicated by pneumothorax, so a chest tube was put into place to treat this complication. We eventually performed pleurodesis, given continued drainage from the chest tube.

**Figure 8:**
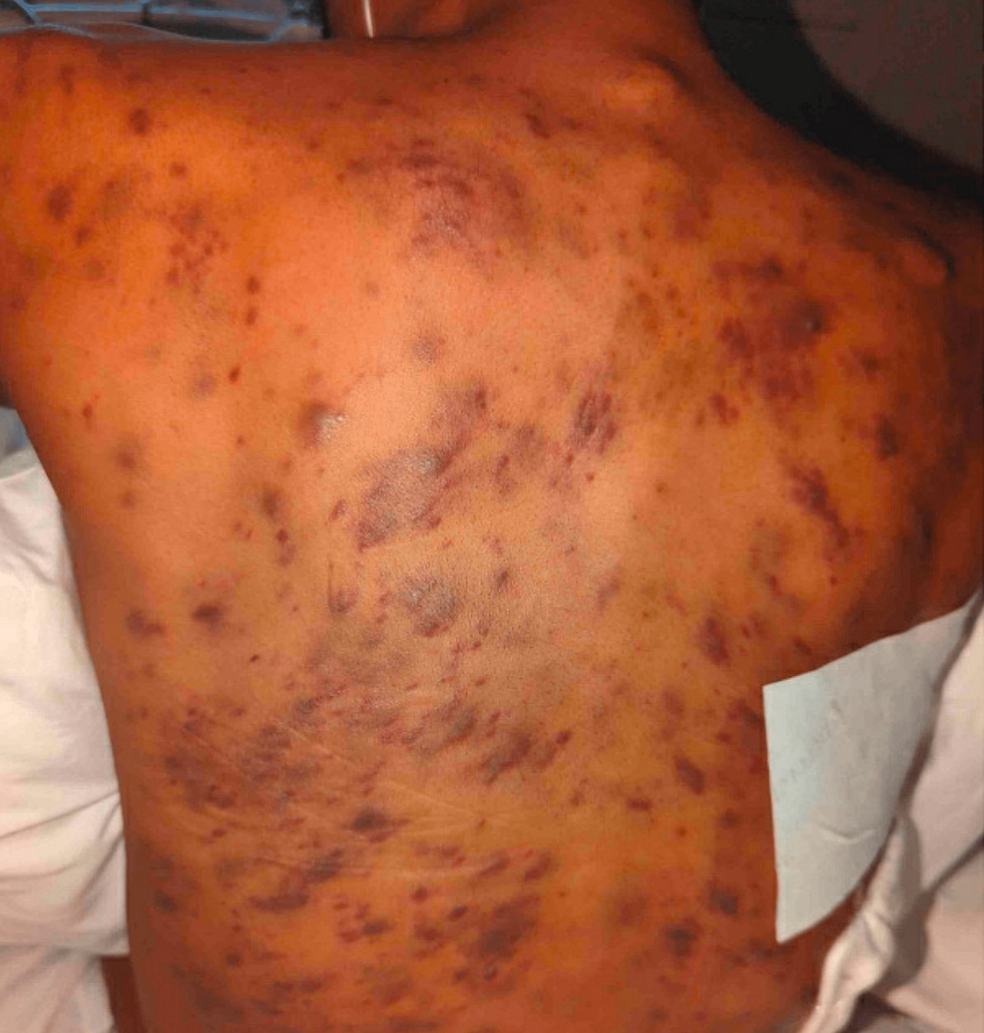
Kaposi sarcoma lesions on the patient's back following doxorubicin therapy.

The patient was continued on his home ART regimen throughout his admission. However, the patient's clinical condition not only failed to improve on ART alone, but continued to worsen; new skin lesions were developing, the edema was causing increasing discomfort, and his shortness of breath was not resolving. He was started on doxorubicin therapy after a thorough discussion with the patient regarding benefits and risks. Following the first cycle of chemotherapy, he had a positive response; his skin lesions and cervical lymph nodes regressed in size (Figure 8). Additionally, there was a significant decrease in swelling of his extremities and genitals.

The patient was discharged home due to clinical improvement. He was connected with home services to assist him with activities of daily living (ADLs) and instrumental ADLs (IADLs) and he was scheduled for routine outpatient follow-up and outpatient chemotherapy.

## Discussion

This case highlights the interesting relationship between pre-existing KS and the development of IRIS and disseminated KS. In one retrospective study, researchers found that patients with KS on presentation had the highest percentage of IRIS following ART initiation compared to other AIDS-defining illnesses [5]. One randomized control trial found that almost 60% of individuals with pre-existing KS developed IRIS after initiating therapy, which can then lead to the rapid expansion of the lesions [6]. KS can disseminate to the musculoskeletal, lymphatic, gastrointestinal, and pulmonary systems, sometimes even in the absence of cutaneous lesions [7]. KS of the respiratory structures has a poor prognosis; one cohort study found that patients with lung involvement had a median survival time of approximately 1.6 years [2].

Interestingly, recent studies investigating outcome predictors in KS showed that interleukin (IL)-5 levels are higher in patients with a positive clinical response to ART (lesion regression), while levels of IL-6 and IP-10 (interferon-gamma inducible protein of 10 kDa) are higher in patients with negative clinical response to ART (lesion progression) [8]. Therefore, healthcare professionals may consider measuring these markers prior to and following ART initiation to supplement their monitoring of treatment response and disease progression in their patients. Additionally, there are treatment options for those with significant KS lesions. A randomized, controlled, open-label trial found that dual treatment with ART and chemotherapy significantly reduced KS lesions within a year [9]. In the patient discussed in the current report, the initiation of chemotherapy showed rapid clinical improvement. Therefore, while this condition may be clinically rare, it has significant clinical outcomes and, therefore, requires the clinician's awareness when treating individuals with HIV. 

## Conclusions

This case report presents several important implications in HIV care. One is that healthcare professionals should carefully screen patients with HIV for opportunistic infections prior to ART initiation. Healthcare professionals should then closely monitor their patients after ART initiation for signs of IRIS, especially in individuals with pre-existing AIDS-defining illnesses such as KS. Both these first two suggestions may also include measurement of IL-5, IL-6, and IP-10 to predict disease outcomes following ART initiation, if clinic or hospital resources allow such. Additionally, it is important to include pulmonary KS in the differential diagnosis for patients with HIV presenting with pulmonary signs and symptoms. This is important since, as mentioned previously, visceral organs may be involved in the absence of skin lesions. Lastly, a diagnosis of disseminated KS may warrant prompt consideration of chemotherapy to prevent the significant morbidity and mortality associated with this condition. 
